# Investigating the Broad Matrix-Gate Network in the Mitochondrial ADP/ATP Carrier through Molecular Dynamics Simulations

**DOI:** 10.3390/molecules27031071

**Published:** 2022-02-05

**Authors:** Shihao Yao, Boyuan Ma, Qiuzi Yi, Min-Xin Guan, Xiaohui Cang

**Affiliations:** 1 Division of Medical Genetics and Genomics, The Children’s Hospital, Zhejiang University School of Medicine, Hangzhou 310052, China; 11618045@zju.edu.cn (S.Y.); 102835@zju.edu.cn (B.M.); 21718643@zju.edu.cn (Q.Y.); 2 Institute of Genetics, and Department of Genetics, Zhejiang University School of Medicine, Hangzhou 310058, China

**Keywords:** ADP/ATP carrier, mitochondrial carrier family, transporter, molecular dynamics simulation

## Abstract

The mitochondrial ADP/ATP carrier (AAC) exports ATP and imports ADP through alternating between cytosol-open (c-) and matrix-open (m-) states. The salt bridge networks near the matrix side (m-gate) and cytosol side (c-gate) are thought to be crucial for state transitions, yet our knowledge on these networks is still limited. In the current work, we focus on more conserved m-gate network in the c-state AAC. All-atom molecular dynamics (MD) simulations on a variety of mutants and the CATR-AAC complex have revealed that: (1) without involvement of other positive residues, the charged residues from the three Px[DE]xx[KR] motifs only are prone to form symmetrical inter-helical network; (2) R235 plays a determinant role for the asymmetry in m-gate network of AAC; (3) R235 significantly strengthens the interactions between H3 and H5; (4) R79 exhibits more significant impact on m-gate than R279; (5) CATR promotes symmetry in m-gate mainly through separating R234 from D231 and fixing R79; (6) vulnerability of the H2-H3 interface near matrix side could be functionally important. Our results provide new insights into the highly conserved yet variable m-gate network in the big mitochondrial carrier family.

## 1. Introduction

Mitochondria, well known as the “powerhouses of the cell”, are major places to synthesize ATP through oxidative phosphorylation (OXPHOS) in eukaryotes. Mitochondria also synthesize heme and steroid hormones, and house multiple important metabolic pathways including tricarboxylic acid cycle, urea cycle and β-oxidation. In addition, as the semi-autonomous organelles, mitochondria need to replicate their own DNA, and transcribe and translate genes they carry. To fulfill these functions, solutes including ions, nucleotides, amino acids, fatty acids and many other important metabolites need to be continuously exchanged between cytoplasm and mitochondrial matrix. Members of the mitochondrial carrier family (MCF) facilitate transport of these substrates across the highly impermeable inner mitochondrial membrane (IMM) [1,2,3].

**Figure 1:**
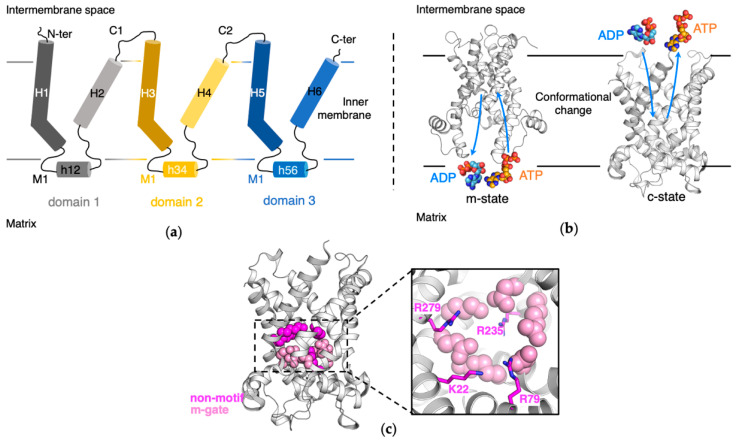
The schematic diagram of AAC’s tripartite structure and function. (**a**) AAC’s tripartite structure. Three domains of AAC are colored in grey, yellow and blue, respectively. (**b**) The schematic diagram of AAC’s function. The c-state (PDB: 1OKC [8]) and m-state (PDB: 6GCI [15]) AAC are shown in cartoon. ADP and ATP are shown in spheres. (**c**) The broad matrix gate in the c-state AAC. The charged residues from the Px[DE]xx[KR] motifs are shown as light pink spheres, and the non-motif charged residues are colored as magenta.

Although substrates transported by mitochondrial carriers (MCs) are extremely diversified, these transporters all have three homologous domains (Figure 1a), and each domain contains many conserved sequence motifs such as [YF][DE]xx[RK], GxxxG and πxxxπ motifs [3] near the cytoplasmic side of the carriers and more conserved MCF motif near the matrix side: Px[DE]xx[KR]xRxxQ-(matrix loop)-[YF]xG-(matrix helix)-[DE]Gxxxx[YWF][KR]G [2,3,4]. The special tripartite symmetry in sequence and highly conserved MCF motif in each homologous domain distinguish this family from other transporter families. Therefore, sequence symmetry analysis is important to understand transport mechanism of this family [5], and residues at the three symmetric positions in the tripartite structure are usually referred to as triplets [6]. Currently, 53 members have been identified in human MCF. This family represents the biggest solute carrier (SLC) subfamily and is also known as SLC25.

Mitochondrial ADP/ATP carrier (AAC, also named ANT for adenine nucleotide translocase) is paradigm of the MCF family and is the most abundant protein in IMM [7]. It imports ADP into the mitochondrial matrix and exports ATP to the cytosol through cycling between the cytosolic-open (c-) state and matrix-open (m-) state upon ligand binding (Figure 1b). AAC is currently the only MCF member whose crystal structures have been resolved. The c-state structure was resolved through co-crystallizing with the specific inhibitor carboxyatractyloside (CATR) [8,9]. The structure exhibits three-fold pseudo-symmetry, with each structurally similar domain containing two transmembrane helices connected by a short amphipathic helix at the matrix end (domain 1: H1-h12-H2, domain 2: H3-h34-H4, domain 3: H5-h56-H6). Proline within the extremely conserved Px[DE]xx[KR] motif introduces a sharp kink in each odd-numbered helix, and the six transmembrane helices surround a big pocket opened towards the cytosol. The six charged residues from the three Px[DE]xx[KR] motifs form a symmetrical and cyclic inter-helical salt bridge network on the matrix side (Figure 1c). This network was called matrix-gate or m-gate [10,11], and it was proposed that the m-gate is an important factor to stabilize the c-state conformation of AAC.

At the m-gate level, other basic residues also appear within the pocket of AAC: K22, R79, R279 and R235, and these residues bind directly or indirectly through water molecules with the inhibitor CATR in the crystal structure. For simplicity, we denote these basic residues in the pocket that do not belong to the Px[DE]xx[KR] motif as “non-motif” basic residues in the current work (Figure 1c), although they may also involve in some motif that has not be recognized. CATR is structurally different and much bulkier than ADP, the natural substrate of c-state AAC. Therefore, it was pointed out that CATR binding might rearrange the salt bridge network and deviate the crystal structure from the natural conformation of either ADP-bound or apo AAC [7]. Various molecular dynamics (MD) simulations on apo c-state AAC did show that the overall salt-bridge network at the bottom of the cavity rearranged after removing CATR [4,12,13,14]. More specifically, our previous simulations on microsecond time scale have shown that when the inhibitor CATR is removed, the charged residues from the Px[DE]xx[KR] sequence mainly form intra-helical salt bridges, and together with polar residues and non-motif positive residues including K79, R235 and R279, form a broad asymmetric electrostatic network at the bottom of the pocket [4]. In this broad asymmetric network, domains 2 and 3 bind strongly together, while domain 1 only binds loosely with the other two domains. Most strikingly, the asymmetry in this broad m-gate network matches with the recently resolved m-state crystal structure in which domain 1 deviates from the other two domains in the matrix side [15]. The partially asymmetric m-state structure was also justified by the asymmetry in the three specific cardiolipin binding sites at the domain-domain interfaces observed in our previous MD simulations [16]. Our previous work implies that m-gate network provides important clues to understand the transport mechanism of AAC.

Our previous results suggest that the non-motif positive residues R79, R235 and R279 are integral part of the broad m-gate network. However, the effect of these residues on the m-gate and the structural dynamics of AAC are unclear. In the current work, we carried out all-atom MD simulations on CATR-bound AAC and the five mutants of apo AAC in which different non-motif positive residues at the m-gate level are mutated to alanine. Through comparing the m-gate network and the structural dynamics of the mutants to those of wild-type apo AAC, we aim to understand how these non-motif residues may contribute to the m-gate profile and affect structural dynamics of the carrier. As CATR binds with both motif and non-motif positive residues, elucidating roles of the non-motif residues on the m-gate network also help clarify how CATR binding may affect this broad network.

## 2. Results

### 2.1. Impact of Mutations of the Non-Motif Positive Residues on the m-Gate Network

**Figure 2:**
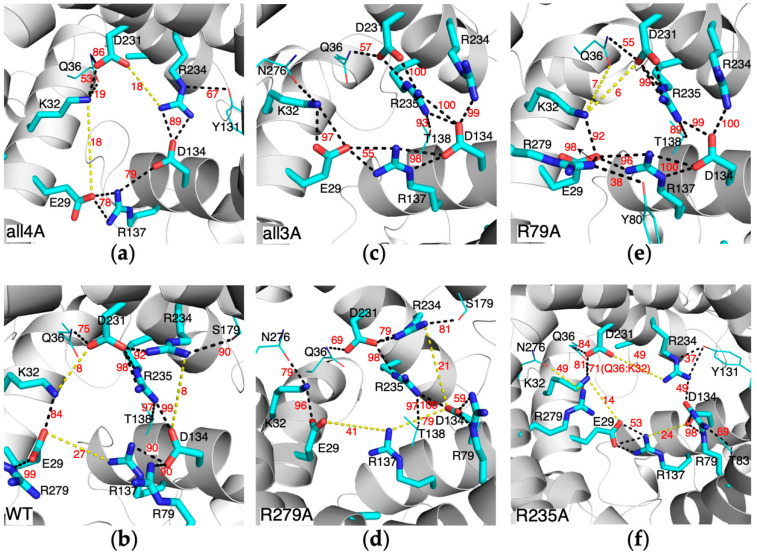
The m-gate electrostatic network in the various mutants (**a**,**c**–**f**) and wild-type (**b**) of apo AAC. Yellow dash lines are added manually to indicate the salt bridges and H-bonds that do not appear in the shown snapshots. Salt bridges or H-bonds with the occupancies lower than 5% are not shown. For mutants, occupancies of salt bridges and H-bonds are calculated on each trajectory from 200 ns to 1 μs. The occupancies in the *wild-type AAC* were averaged over last 2 μs of the three 3-μs trajectories.

To represent the ground state of the m-gate network formed by the charged residues of Px[DE]xx[KR] motif, we built *all4A-AAC* mutant in which all the non-motif positive residues of AAC including K22, R79, R235 and R279 were mutated to alanine. MD simulation on this mutant shows that the charged motif residues are more prone to form inter-helical salt bridges than intra-helical salt bridges (Figure 2a and Figure S1a). The occupancies of the inter-helical salt bridges E29:R137, D134:R234 and D231:K32 are 78%, 89% and 53%, respectively, and the intra-helical salt bridges E29:K32, D231:R234 and D134:R137 show occupancies of 18%, 18% and 79%, respectively. Our results demonstrate that compared to the *wild-type AAC* (Figure 2b and Figure S1b), the ground state of the m-gate network is more symmetrical and contains more inter-helical interactions.

To evaluate how R235 affects the ground state of the m-gate network, we built *all3A-AAC* mutant system in which all the four non-motif positive residues except R235 were mutated to alanine, and this can also be viewed as introducing R235 to *all4A-AAC*. R235 is the featured residue in AAC and the closely related mitochondrial carriers SLC25A16 (Solute carrier family 25, member 16, graves disease carrier) and SLC25A42. We previously reported the central role of R235 in the asymmetric electrostatic network of the *wild-type AAC* [4]. By comparing the simulation results of *all4A-AAC* and *all3A-AAC*, we clearly demonstrate how the presence of R235 dramatically changes the m-gate network (Figure 2c and Figure S1c). In *all3A-AAC*, R235 forms very strong salt bridges with both D134 and D231. This destroys the inter-helical salt bridge D231:K32, while the intra-helical E29:K32 is dramatically strengthened (occupancy: 97%). Meanwhile, R234 forms π-π stacking interaction with R235, which destroys intra-helical salt bridge D231:R234 and strengthens inter-helical salt bridge D134:R234. It’s apparent that the presence of R235 greatly strengthens both intra-helical salt bridges within H3 or H5 and an inter-helical salt bridge between them, while the inter-helical salt bridges between H1 and the other two helices are dramatically weakened. Therefore, our results suggest that R235 is the key determinant for the high asymmetry in the m-gate network.

To assess the effect of introducing R79 to the above *all3A-AAC* mutant, the *R279A-AAC* mutant was built in which both R279 and K22 of AAC were mutated to alanine. The MD simulations show that the presence of R79 lifts up D134 through forming the D134:R79 salt bridge. This does not change the strong D134:R235 salt bridge but deteriorates the D134:R234 salt bridge. R234 changes the orientation to bind with D231 and S179 simultaneously (Figure 2d and Figure S1d). The conformation of R234 in *R279A-AAC* is quite similar to that in the *wild-type AAC* (Figure 2b and Figure S1b). Meanwhile, the effect of R79 on the matrix-gate network can also be evaluated by comparing the simulations results of *wild-type AAC* and *R79A-AAC* in which both R79 and K22 were mutated to alanine. Compared to *R79A-AAC*, in *wild-type AAC* the conformation of R234 is drastically changed and the inter-helical D134:R234 salt bridge is destroyed, with occupancy decreased from 100% to 8%. In addition, the inter-helical E29:R137 salt bridge is disrupted and the occupancy dropped from 96% to 27%. Therefore, comparisons between these two pairs of systems lead to consistent results on the effect of R79 to the m-gate network. In brief, R79 drastically deteriorates the inter-helical salt bridge D134:R234 and E29:R137.

To estimate the impact of introducing R279 to the above *all3A-AAC* mutant, the *R79A-AAC* mutant was built in which both R79 and K22 of AAC were mutated to alanine. The MD simulations show that R279 forms a stable salt bridge with E29, but it does not bring obvious changes to the m-gate network except that the inter-helical E29:R137 salt bridge is strengthened (occupancy: 96%) (Figure 2e and Figure S1e). Comparing the simulations results on *wild-type AAC* and *R279A-AAC* leads to a similar conclusion: R279 does not drastically change the m-gate network.

In *R235A-AAC*, R235 is mutated to alanine while all the other non-motif charges residues (K22, R79 and R279) were maintained. MD simulations show that in this mutant, R79 forms a very strong salt bridge with D134 and lifts up D134 which weakens both intra-helical salt bridge D134:R137 and inter-helical salt bridge D134:R234. Here, R279 only binds with N276 and does not become involved in the m-gate network. Basically, the m-gate network in R235A-AAC is quite similar to that in all4A-AAC, which is more symmetrical and contains more inter-helical populations. This again highlights the determinant role of R235 in the asymmetry of the m-gate network of AAC. Moreover, whenever R235 appears, the salt bridge connecting H3 and H5 exhibits extremely high occupancy of almost 100% (Figure 2b–e and Figure S1b–e). In contrast, the salt bridge between H3 and H5 (D134:R234) shows an occupancy of 89% in *all4A-AAC* and occupancy of 49% in *R235A-AAC*. These results demonstrate that R235 dramatically strengthen the interactions between H3 and H5.

### 2.2. Impact of CATR Binding on the m-Gate Network

**Figure 3:**
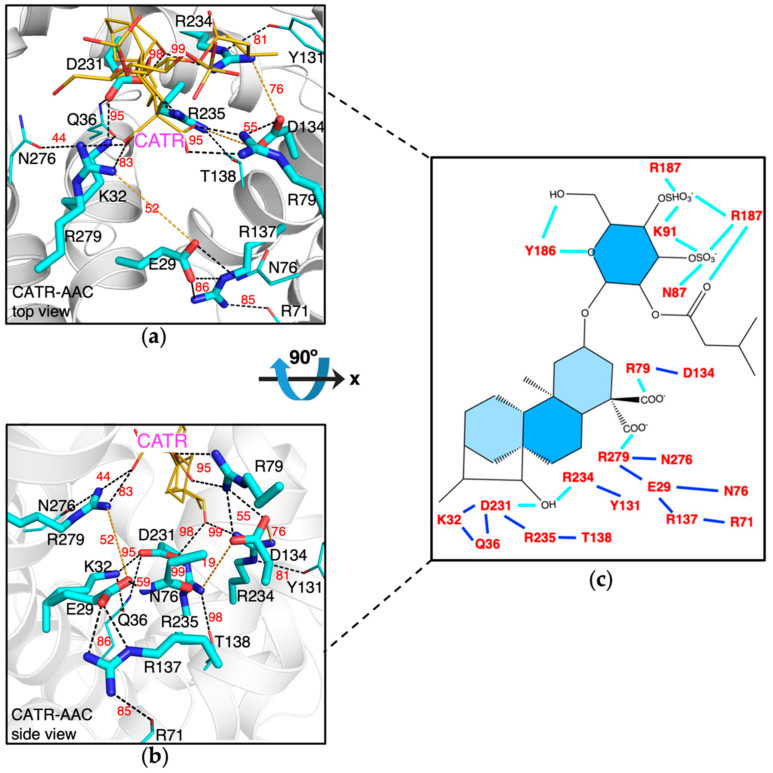
The m-gate electrostatic network in the AAC-CATR complex. Top view (**a**) and side view (**b**) of the AAC-CATR interactions in the last simulation snapshot (1 μs). CATR is shown in yellow think sticks. In the side-view figure, protein backbones are shown in transparent cartoon. Yellow dash lines are added manually to indicate the H-bonds that do not appear in the shown snapshot. Q36 forms H-bond with both D231 (occupancy: 71%) and K32 (occupancy: 94%), and these values are not labeled in the figure because of limited space. The H-bonds with the occupancies lower than 5% are not shown. The occupancies are calculated on the trajectory from 200 ns to 1 μs. (**c**) A schematic representation of the CATR-AAC interactions. The electrostatic interactions between CATR and AAC are shown in cyan lines, and the interactions within the m-gate network are shown in blue lines.

To investigate the effect of CATR binding to the m-gate network, we ran 1-μs MD simulation on ACC in complex with CATR, and compared the results to those of the wild-type apo AAC and the mutants described above. The m-gate network in the crystal structure is well maintained in the simulation on the *CATR-AAC* complex (Figure 3). The central hydroxyl group of CATR forms H-bonds to both D231 and R234 on H5 with high occupancies of 98% and 99%, respectively, and this effectively prevents the formation of intra-helical R234:D231 salt bridge (Figure 3). Moreover, the intervention of the central hydroxyl group of CATR between D231 and R234 pushes D231 closer to H1 and R234 closer to H3, which favors the formation of the inter-helical salt bridges D231:K32 (occupancy: 95%) and D134:R234 (occupancy: 76%), respectively. In fact, D231 forms extensive interactions with K32, Q36, R235 and the central hydroxyl group of CATR, which effectively prevents the formation of the intra-helical E29:K32 salt bridge. In addition to binding with D231 with high occupancy of 99%, R235 also binds with T138 with high occupancy 98%. R235 only binds with D134 occasionally with low occupancy of 19%. R79 binds to the primary carboxyl group of CATR (the only carboxyl group of ATR). In fact, this carboxyl group together with the central hydroxyl group of CATR forms an extensive electrostatic network with R234, Y131, D134 and R79 as in the crystal structure, and this effectively prevents D134 from forming intra-helical salt bridge with R137. This leads to the high occupancy of the inter-helical salt bridge R137:E29 of around 86%. Although R279 is at the equivalent position of R79, it binds to the secondary carboxyl group of CATR a bit more dynamically than R79. In summary, our simulations on the CATR-AAC complex, together with the simulation results on the above mutants, reveal the effect of CATR binding to the m-gate network: CATR promotes symmetry and inter-helical salt bridges in the m-gate network mainly by separating R234 from D231 with its central hydroxyl group and through fixing R79 with its primary carboxyl group. Our results are in agreement with the ability of CATR to lock AAC in the c-state conformation.

### 2.3. The Conserved Gap between A26 and the [YWF][KR]G Motif in Domain 1

**Figure 4:**
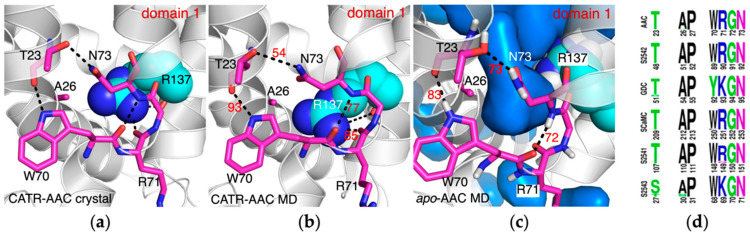
Conformations of R137 are quite different with and without presence of CATR. In the crystal structure (**a**) and the MD simulation structure (**b**) of CATR-AAC complex, the guanidine group of R137 binds with backbone carbonyl of R71 and fills the gap between the [YWF][KR]G motif and A26. (**c**) The gap between the [YWF][KR]G motif and A26 is filled with water molecules in the simulations on apo AAC (*wild-type AAC*). (**d**) Sequence logo of A26 and residues involved in the interaction between the [YWF][KR]G motif and Pro kink region in orthologs of AAC, SLC25A42, GDC (graves disease carrier), SCaMC (calcium-binding mitochondrial carrier protein), SLC25A41 and SLC25A43, respectively. Equivalent positions among these paralogs are aligned in the same column. The multiple sequence alignment of SLC25A43 was calculated based on 171 sequences from the UniProt database. The results of other carriers were obtained from our recent work [17]. The residues are numbered based on hAAC1 (human ADP/ATP carrier), SLC25A42, SLC25A16, SCaMC1, SLC25A41 and SLC25A43, respectively.

When R137 forms a salt bridge with E29 in the crystal structure or in MD simulations of the CATR-AAC complex, it forms H-bond with the backbone carbonyl group of R71, the middle residue of the conserved [YWF][KR]G motif in domain 1. In this way, the guanidine group of R137 fills the gap formed between the β-turn structure of the [YWF][KR]G motif and the small residue A26 (Figure 4a,b). In contrast, without the presence of CATR in the simulations of apo AAC, R137 moves up to bind with D134, and the gap is filled with solvent molecules (Figure 4c). Here the solvent in the pocket is very close to the solvent from the matrix side, and the only barrier between them is the β-turn structure of the [YWF][KR]G motif. This implies that, due to the presence of A26, the interface of the matrix ends of H2 and H3 could be more vulnerable to ligand binding in the pocket.

### 2.4. Impact of Mutations of the Non-Motif Positive Residues on the Structural Dynamics of AAC

**Figure 5:**
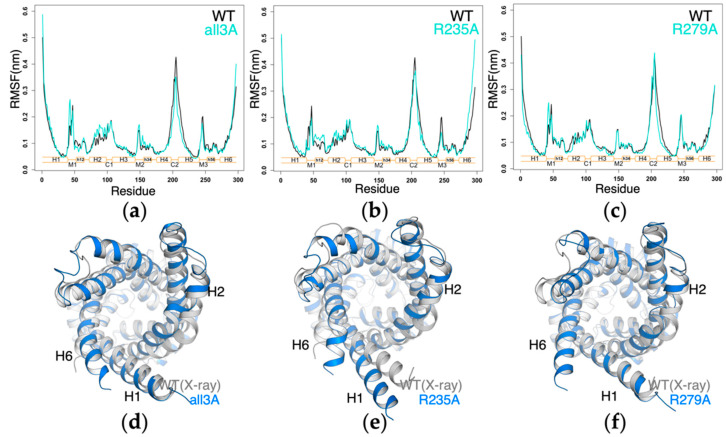
Structures and dynamics of AAC were not significantly affected in the *all3A-AAC*, *R235A-AAC* and *R279A-AAC* mutants. (**a**–**c**) RMSF of three mutants compared to that of *wild-type AAC*. The RMSF were calculated on each trajectory from 200 ns to 1 μs. (**d**–**f**) Superposition of the structures at the end of the simulation (1 μs) to the crystal structure (PDB:1OKC).

To further investigate the influence of the above mutations on the structure and dynamics of AAC, we compared the root mean square fluctuation (RMSF) of each mutant system with *wild-type AAC*, and superimposed the last snapshot of each simulation on the crystal structure. The 1-μs simulation results show that the *all3A-AAC*, *R235A-AAC* and *R279A-AAC* show quite similar structural dynamics as the *wild-type AAC* (Figure 5a–c). The crystal structure conformation is well maintained in these three mutants except that the inward movement of H2 was consistently observed (Figure 5d–f). In *R235A-AAC*, a drift of the cytoplasmic half of H1 and backbone bending near the H6 terminal end were also observed (Figure 5e). The bending of H6 was also reported in one simulation of the *wild-type AAC* on a longer time scale [4].

**Figure 6:**
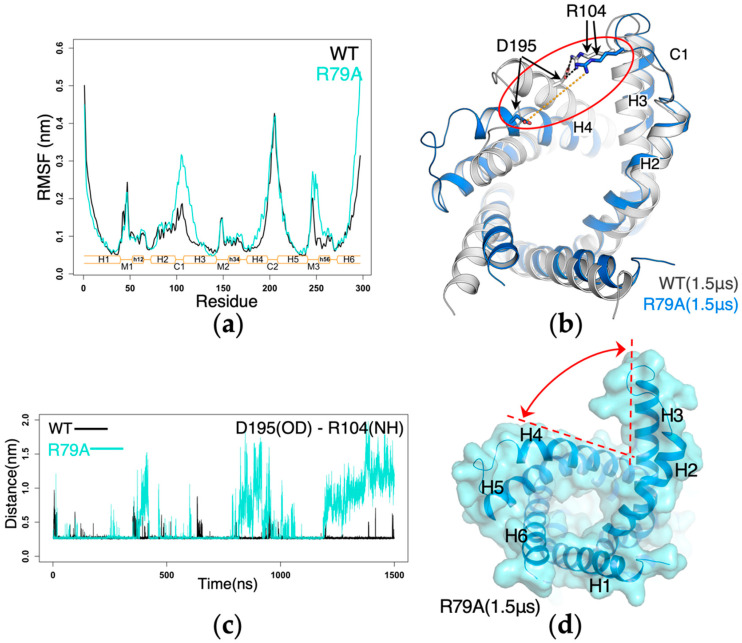
The R79A mutation causes drastic conformational change around C1 loop. (**a**) RMSF of the mutant compared to that of *wild-type AAC*. The RMSF were calculated on each trajectory from 200 ns to 1 μs. (**b**) Superposition of the structures at the end of the simulations of *R79A-AAC* and *wild-type AAC* (1.5 μs). (**c**) Time evolution of the minimum distance between R104 and D195 heavy atoms during the simulations. (**d**) A big crevice formed between the cytoplasmic halves of H3 and H4.

In *R79A-AAC*, significant structural changes were observed at the cytoplasmic side, so we extended the simulation to 1.5 μs. In *wild-type AAC*, C1 loop is much more stable than C2 loop as reflected by the much lower RMSF values (Figure 6a), and a major stabilizing factor is the strong D195(H4):R104(C1 loop) salt bridge that attaches C1 loop to H4 (Figure 6b). This salt bridge is extremely stable in the simulations of *wild-type AAC*, but it became much more dynamic in *R79A-AAC* (Figure 6c). Therefore, the C1 loop together with cytoplasmic sides of H2 and H3 helices are much more flexible in *R79A-AAC* as reflected by the dramatic increase in the RMSF values of this region (Figure 6a). Starting at 1193 ns, the dynamic R104:D195 salt bridge completely separated (Figure 6b), forming a big crevice between the cytoplasmic halves of the H3 and H4 helices in *R79A-AAC* trajectory (Figure 6d). Moreover, drastic and dynamic distortions are observed within the helix backbone of H3, which is also reflected by the big difference of RMSF values for the H3 region in *R79A-AAC* and in *wild-type AAC* (Figure 6a).

**Figure 7:**
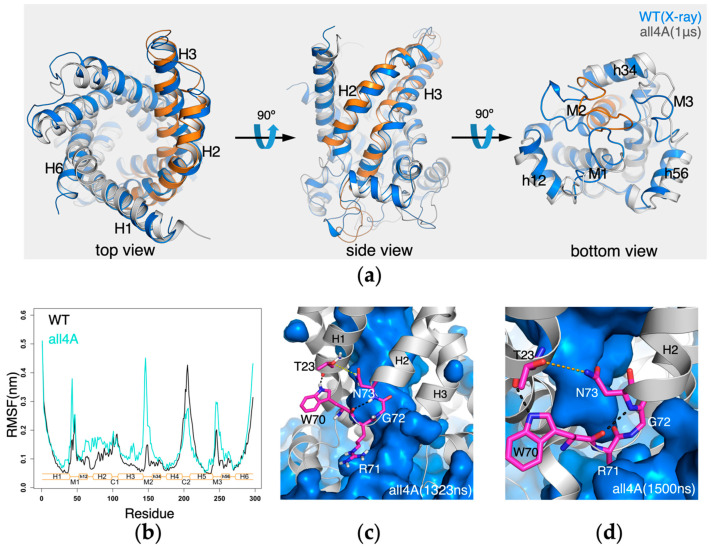
Changes in the structure and dynamics in *all4A-AAC*. (**a**) Superposition of the structures at the end of the simulation (1 μs) to the crystal structure (PDB: 1OKC). The H2, C1 loop, H3 and M2 loop of the simulated structure are present in cartoon mode and highlighted in orange. (**b**) RMSF of *all4A-AAC* compared to that of *wild-type AAC*. The RMSF were calculated on each trajectory from 200 ns to 1 μs. (**c**) A crevice formed between the matrix end of H3 and the β-turn structure of the [YWF][KR]G motif which allows for the passing through of the solvent. (**d**) The crevice between H2 and H3 interface became closed after transient opening.

In *all4A-AAC*, significant structural changes occurred at the matrix side, more specifically, in the M1 loop, M2 loop and the matrix halves of H2 and H3 helices (Figure 7a). When the RMSF curves of *all4A-AAC* and *wild-type AAC* are compared, a most significant difference is observed in the M2 loop (Figure 7b). In the crystal structures of bovine AAC1, the guanidinium groups of R30, R71 and R151 form a nice stacking structure. In the simulations of *all4A-AAC*, the R30:R71:R151 stacking structure became dissociated at 372 ns, and this led to the disruption of the electrostatic interaction between R30 and N-terminus of H3 and also the cyclic electrostatic network (Figure S3). In contrast, although the R30:R71:R151 stacking structure also become dissociated with longer simulation time (at around 1.3 μs) in *wild-type* AAC, the dissociation of the stacking structure did not lead to the disruption of the cyclic electrostatic network between capping arginines and N-termini of odd-numbered helices (Figure S3). The difference highlights the significance of the non-motif positive residues in maintaining the structure of the c-state AAC. Meanwhile, in *all4A-AAC* a narrow crevice quickly formed at 380 ns between the matrix ends of H2 and H3, and through this crevice solvent in the pocket went through with the bulk solvent at the matrix side. This narrow crevice became closed at 430 ns, and from 850 ns an even bigger crevice formed at the same interface till the end of the simulation (Figure 7c). These results support the above speculation on the vulnerability of the H2-H3 interface inferred from the simulation results of *wild-type AAC*, and also suggest the stabilization role of the R30:R71:R151 stacking structure on the vulnerable H2-H3 interface. To investigate whether the big crevice at the H2-H3 interface will lead to more drastic conformational changes to the carrier, we extended the 1-μs simulation of *all4A-AAC* for additional 500 ns. Molecular dynamic simulation results show that the opening of the H2-H3 interface did not lead to more drastic conformational changes, instead, the crevice became closed just before the end of the 1.5-μs simulation (Figure 7d). Results show a strong inclination of AAC to maintain its intact ground c-state conformation, and its transition to the m-state can only be triggered upon ligand binding.

### 2.5. Structure-Based Symmetry Analysis near the m-Gate Level

**Figure 8:**
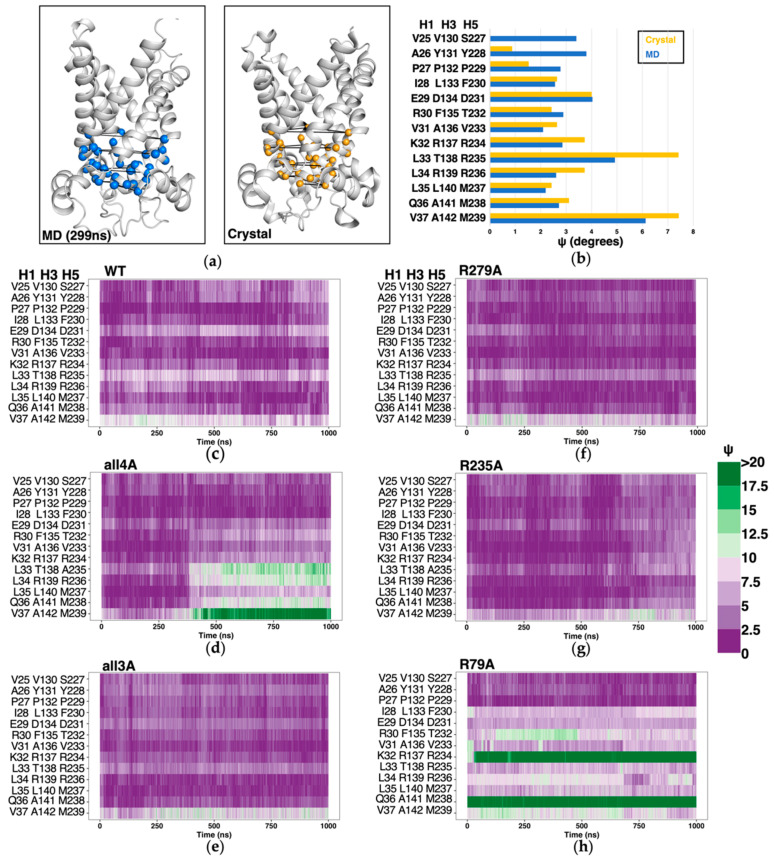
Geometrical symmetry around the m-gate level in c-state AAC. (**a**) The model used to calculate deviations from the C_3_-symmetry score. The triplets used for calculation are shown in spheres. (**b**) The average ψ values of the *wild-type* simulation structure and crystal structure of c-state AAC (PDB: 1OKC). (**c**–**h**) Time evolution of the geometric symmetry analysis in *wild-type AAC* and the five mutants. Gradient colors are used to indicate the extent to which each plane deviates from C_3_-symmetry.

Threefold pseudo-symmetry is an important feature of the c-state structure of AAC, and investigations on the relationship between m-gate and structural symmetry near the m-gate level might provide more clues to understand the transport mechanism of AAC. In the previous work on m-state AAC, deviation of each triangle formed by three Cα atoms of each triplet group from an equilateral triangle was used to assess the degree of geometrical symmetry, and more specifically, the metric ψ was calculated through averaging the deviations of the three triangle angles from 60° [18]. Higher values of ψ indicate a high deviation from C_3_ symmetry for the triplet residues. Following the same method, here we analyzed symmetry degree in H1, H3 and H5 near the m-gate level, ranging from triplets 37 to triplets 25 (Figure 8a). We first calculated ψ values for the CATR-inhibited crystal structure (PDB: 1OKC), and compared to the averaged ψ values based on 3 μs MD simulation trajectory of *wild-type AAC* (Figure 8b). The results show that in the simulations on *wild-type AAC*, triplets 31 to 37 consistently exhibit lower ψ values in simulated apo AAC than in CATR-bound crystal structure, while most of the preceding triplets (triplets 25 to 30) are more symmetrical in the CATR-bound crystal structure than in apo AAC. Due to different progression directions of the three matrix loops, the triplet 37 at the terminal ends of the odd-numbered helices has a very high ψ value in both the crystal structure and in apo AAC. Worthy of special mentioning, the triplet 33 (L33, T138 and R235) also exhibits a much higher deviation in symmetry than the neighboring triplets, and this is consistent with the determinant role of R235 in the asymmetry of the m-gate network as described above. Then we calculated the time evolutions of ψ values of the triplets around the m-gate level in different simulation systems (Figure 8c–h). Compared to *wild-type AAC* (Figure 8c), geometrical symmetry in *R279A-AAC* and *R235A-AAC* systems (Figure 8f,g) is generally improved. Of special interest, in *all4A-AAC*, high deviation value was observed after 300 ns (Figure 8d), although the m-gate network in this mutant is more symmetric and has more inter-helical interactions than that in the *wild-type AAC* (Figure 2a,b). This result indicates the importance of the non-motif charged residues in maintaining the geometrical symmetry, although these non-motif residues are asymmetrically distributed. Most significantly, the R79A mutation severely deteriorates the geometrical symmetry around the m-gate level since the beginning of the simulation, especially for the triplets 32 and 36 (Figure 8h), and this is mainly caused by the backbone distortions within H3. This result highlights the significance of R79 in maintaining the structural integrity and symmetry of AAC.

## 3. Discussion

**Figure 9:**
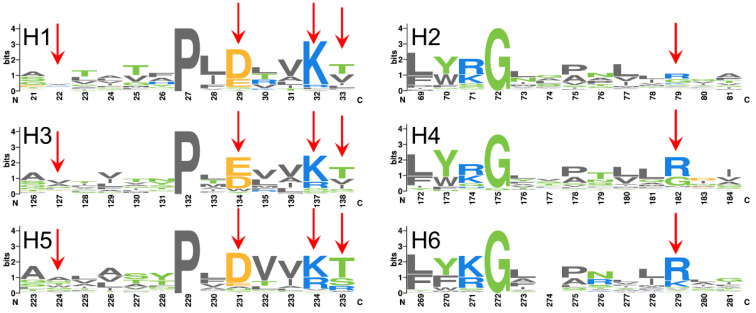
Sequence logo presentation of 53 human mitochondria carriers. Only residues around the m-gate level in odd-numbered helices (**left**) and even-numbered helices (**right**) are shown. Triplet positions at least with one charged residue in AAC are highlighted with red arrows.

Compared to most membrane proteins, mitochondrial carriers are featured with significantly large positive net charges [12]. Within the pocket, in addition to the balanced charges from the Px[DE]xx[KR] motif on odd-numbered helices, positive residues also appear at high frequencies at positions equivalent to the triplet 79 of AAC (Figure 9) on even-numbered helices, and this leads to the net positive charges near the bottom of the pocket. Considering consensus sequence feature at the triplet 79 positions in contrast to the tremendous diversity in structures and charges of the substrates transported by mitochondrial carriers, we infer that the triplet 79 positions could be more related to the general transport mechanism shared by this family, rather than carrying out substrate discrimination function. This inference is also supported by our recent identification of a new highly specific ADP binding site near the upper region of the cavity in c-state AAC [17].

In AAC, in addition to R79 and R279 at the triplet 79 positions, R235 within the featured RRRMMM motif also appear at the bottom of the cavity. Our previous work have shown that without presence of the inhibitor CATR, these positive residues together with charged residues of the Px[DE]xx[KR] motifs form a broad asymmetric m-gate network [4]. The difference between the m-gate networks with and without presence of CATR was also highlighted in earlier MD simulation studies on AAC [13,19]. A recent study on the uncoupling protein UCP2 also demonstrated the engagement of the three triplet 79 residues (R88, R185 and R279) in the broad matrix network [20]. Therefore, results from different groups consistently suggest that these non-motif basic residues are integral part of the broad matrix network. In the current work, through MD simulations on a variety of AAC mutants, contributions from these non-motif positive residues on the profile of m-gate network and structural dynamics of AAC were assessed.

Our findings suggest that R235 is the key residue that determines the asymmetry of the m-gate network, and it also dramatically strengthens the interactions between H3 and H5 (Figure 2 and Figure S1). In the partially asymmetric crystal structure of m-state AAC, domain 2 and domain 3 still attach together at the matrix side when domain 1 separates from these two domains. This implies the importance of R235 in holding domains 2 and 3 together during state transitions. In the previous studies, R253I mutation in yeast AAC2 results in loss of function of the carrier (corresponding to R235 in bovine AAC1) [21,22].

Although R79 and R279 are at equivalent positions in homologous domains 1 and 3, they play quite different roles in affecting the m-gate network. Our results show that through forming a salt bridge with D134, R79 promotes intra-helical D231:R234 and weakens inter-helical salt bridges D134:R234 and E29:R137. Although R279 also forms a stable salt bridge with E29 in both *wild-type AAC* and *R79A-AAC*, R279 does not significantly change the m-gate network, which is different from our previous speculation [4]. The less impact of R279 on the m-gate network is consistent with the previous experiment in which the R279A mutant still retained good transport activity and high expression level, while R79A mutation led to loss of function of the carrier [22]. R79H mutation was also reported to cause severe early-onset dominant mitochondrial disease [23].

Our results suggest that the inhibitor CATR promotes symmetrical m-gate network mainly through separating R234 from D231 and fixing R79. CATR binds with R79 through its primary carboxyl group that is also shared by atractyloside (ATR). The less impact of R279 on m-gate network also helps explain why ATR that does not have secondary carboxyl group to bind with R279 can still stabilize and inhibit the c-state AAC effectively.

K22 is also an important positive residue within the pocket of AAC and its physiological significance has been confirmed by mutagenesis [21] and acetylation experiments [24,25]. However, K22 does not become involved in forming salt bridges with other residues in *wild-type* apo AAC [4], and therefore we did not investigate the impact of K22 in the current work. MD simulations on ADP binding process suggest that K22 might help catch the ADP detached from the specific binding site and relay it to the central binding site [17].

The current work revealed some special structural elements near the matrix ends of H2 and H3. Presence of a small alanine residue A26 before the conserved kink proline P27 makes the β-turn structure of the [YWF][KR]G motif the only barrier between the solvent in the pocket and the bulk solvent in the matrix side (Figure 4c), and hence the interface between the matrix ends of H2 and H3 is speculated to be more vulnerable upon ligand binding. The vulnerability of this interface was further confirmed by the simulation on the *all4A-AAC* mutant (Figure 7d). Residues involved in this interface (A26, T23, W70, R71, G72 and N73) are extremely conserved in both orthologs and paralogs of AAC, SLC25A42, GDC, SCaMC, SLC25A41 and SLC25A43 (Figure 4d). This implies that vulnerability of this interface between domains 1 and 2 could be important for the common transport mechanism of these adenine nucleotide transporters. 

Our previous work showed that due to the dynamic property of H6, the [YWF][KR]G motif in domain 3 lost its β-turn structure in one of the three 3-μs trajectories on *wild-type* apo AAC, and Y228 before the kink proline P229 becomes the only barrier to separate the solvent in the pocket from the bulk solvent in the matrix side [17]. This observation also indicates the vulnerability of the interface between domains 3 and 1. On the other hand, presence of R235 significantly strengthens the H3–H5 (domains 2–3) attachment as mentioned above, and moreover, a stable aromatic cluster forms between the [YWF][RK]G motif and Pro kink region in domain 2 [4]. These interactions separate the solvent in the pocket far away from the matrix solvent near the interface between domains 2 and 3 (Figure S2a). In addition, our previous work also shows that the bound cardiolipin at the specific site of domain 2–3 interface predominantly adopts the inter-domain binding mode [16]. Therefore, all these simulation results are quite consistent with and also justify the partially asymmetric crystal structure of m-state AAC, in which domains 2 and 3 attach together, while domain 1 separates from the other two domains.

The transient opening between β-turn structure of the [YWF][KR]G motif of domain 1 and the matrix end of H3 in *all4A-AAC* is currently the only structural changes we’ve observed through which solvent in the pocket of AAC can go through to the bulk solvent in the matrix (Figure 7d). A recent study reported that H^+^ transport is an integral function of AAC [26]. We speculate that H^+^ could possibly be transported through this transiently opened crevice between the matrix ends of H2 and H3 in c-state AAC even without presence of substrate binding.

The current work is limited in that the simulation time of 1 μs or 1.5 μs is still too short to sample enough conformational space of AAC. Although the *all3A-AAC*, *R235A-AAC* and *R279-AAC* mutants maintained the crystal structure conformation in the 1-μs simulations, this does not mean that the structure could be stably maintained on longer time scale. Moreover, to observe more drastic conformational changes in the limited simulation time, we used the simplified POPC lipid bilayer without presence of cardiolipins in both wild-type and mutant AAC systems, which may cause some artifacts to the m-gate networks reported here. It will be interesting to elucidate how cardiolipins will affect the m-gate network and structural dynamics of AAC in future studies.

## 4. Materials and Methods

### 4.1. System Setup

Initial coordinates for the five AAC mutant systems (*all4A-AAC*, *all3A-AAC*, *R279A-AAC*, *R79A-AAC*, *R235A-AAC*) and the *CATR-AAC* complex system were built from the coordinates of the wild-type apo AAC system after the second equilibration step in our previous work [4]. In the second equilibration step of that work, we used up to 160 ns to fully equilibrate the lipid bilayer and the solvent, with positional restraints applied on heavy atoms of the carrier, and the wild-type AAC system is composed of 70,769 atoms, including one AAC molecule, 219 POPC(palmitoyl-oleoyl-phosphatidylcholine) lipids, 1,2226 water molecules, 23 Na^+^ and 42 Cl^-^. Please refer to the work for more details for system setup, energetic minimization and equilibration [4].

For the AAC mutants, the non-motif positive residues of AAC were mutated to alanine in PyMOL [27] (Table 1), with the coordinates of lipids and solvent kept from the equilibrated wild-type AAC system, and the reduced positive charges caused by mutations were balanced through removing equal number of sodium ions from the system. To set up the *CATR-AAC* system, we fitted the crystal structure of AAC that is bound with CATR (PDB: 1OKC) [8] to the carrier in the equilibrated system to obtain coordinates of CATR, and we removed the water molecules in the original system that are overlapped with CATR.

### 4.2. MD Simulation Protocol

MD simulations were carried out with the GROMACS 4.5.5 package [28] in periodic condition, with the CMAP modified CHARMM36 force field [29,30] applied to the protein and the CHARMM lipid parameters used for POPC [31].After the above systems were setup, each system was first heated from 50 K to 310 K in NBV ensemble with positional restraints applied on all heavy atoms of the protein, and time step was set to 2 fs. In the second and third steps, positional restraints were applied on main-chain atoms and Cα atoms of the protein, respectively, and each step lasts 10 ns. After equilibration, a 1-μs production simulation follows, and for some mutants the simulation was extended to 1.5 μs. The temperature of the system was maintained at 310 K with the v-rescale method [32], with the coupling time of 0.1 ps. The pressure was maintained at 1 bar using the Berendsen method [33] with τ_p_ of 1.0 ps and compressibility of 4.5 × 10^−5^ bar^−1^. The SETTLE [34] and LINCS constraints [35] were applied on the hydrogen-involved covalent bonds in water molecules and in other molecules, respectively. Electrostatic interactions were calculated with the Particle-Mesh Ewald (PME) algorithm [36]. The coordinates of each system were saved every 10 ps. Most analyses were carried out with programs provided in GROMACS package. Trajectories were viewed with VMD [37], and structural graphics were prepared with PyMOL [27].

## 5. Conclusions

In the current work, MD simulations on a variety of AAC mutants have demonstrated that: (1) without presence of non-motif positive residues, the charged residues of the PX[DE]XX[KR] motif are prone to form symmetrical inter-helical m-gate network; (2) R235 plays a determinant role in the asymmetry of the m-gate network of AAC; (3) R235 dramatically strengthens the interactions between H3 and H5; (4) R79 promotes intra-helical D231:R234 and weakens inter-helical salt bridges D134:R234 and E29:R137; (5) R279 does not significantly change the profile of the m-gate network. Our simulation results also suggest that the inhibitor CATR promotes symmetrical m-gate network mainly through separating R234 from D231 and fixing R79. Although limited in the length of simulation time, significant changes in the structural dynamics were observed in the *all4A-AAC* and *R79A-AAC* mutants. The current work highlights that the non-motif positive residues are integral part of the broad m-gate network, and they play different roles in shaping the profile of the broad m-gate network.

## Figures and Tables

**Table 1:** Summary of simulations in the current work.

System	Mutations	Simulation Time
*all4A-AAC*	K22A + R79A + R235A + R279A	1.5 μs
*all3A-AAC*	K22A + R79A + R279A	1 μs
*R279A-AAC*	K22A + R279A	1 μs
*R235A-AAC*	K22A + R235A	1 μs
*R79A-AAC*	K22A + R79A	1.5 μs
*CATR-AAC*	/	1 μs

## Data Availability

Data is contained within the article or Supplementary Material.

## Supplementary Materials

The following are available online: Figure S1: The time evolution of the electrostatic interactions within the broad m-gate network in MD simulations on wild-type and mutant AAC. Figure S2: The solvent within the pocket and bulk solvent at the matrix side is more separated due to presence of a tyrosine before the kink proline in domain 2 and domain 3. Figure S3: The R30:R71:R151 stacking structure and time evolution of electrostatic interactions between the capping arginines and N-termini of odd-numbered helices.

## Sample Availability

Samples are not available from the authors.

## Abbreviations

AAC	ADP/ATP carrier
ATR	atractyloside
BKA	bongkrekic acid
CATR	carboxyatractyloside
GDC	graves disease carrier
hAAC1	human ADP/ATP carrier 1
IMM	the inner mitochondrial membrane
m-gate	matrix gate
MCF	mitochondrial carrier family
MCs	mitochondrial carriers
MD	molecular dynamics
OXPHOS	oxidative phosphorylation
POPC	palmitoyl-oleoyl-phosphatidylcholine
RMSF	root-mean square fluctuation
SCaMC	calcium-binding mitochondrial carrier protein
SLC	solute carrier
SLC25A16	Solute carrier family 25, member 16

## References

[1] Aquila H., Link T.A., Klingenberg M. (1987). Solute carriers involved in energy transfer of mitochondria form a homologous protein family. FEBS Lett..

[2] Palmieri F. (1994). Mitochondrial carrier proteins. FEBS Lett..

[3] Ruprecht J.J., Kunji E.R.S. (2021). Structural Mechanism of Transport of Mitochondrial Carriers. Annu. Rev. Biochem..

[4] Yi Q., Li Q., Yao S., Chen Y., Guan M.X., Cang X. (2019). Molecular dynamics simulations on apo ADP/ATP carrier shed new lights on the featured motif of the mitochondrial carriers. Mitochondrion.

[5] Robinson A.J., Overy C., Kunji E.R. (2008). The mechanism of transport by mitochondrial carriers based on analysis of symmetry. Proc. Natl. Acad. Sci. USA.

[6] Pierri C.L., Palmieri F., De Grassi A. (2014). Single-nucleotide evolution quantifies the importance of each site along the structure of mitochondrial carriers. Cell. Mol. Life Sci..

[7] Klingenberg M. (2008). The ADP and ATP transport in mitochondria and its carrier. Biochim. Biophys. Acta.

[8] Pebay-Peyroula E., Dahout-Gonzalez C., Kahn R., Trezeguet V., Lauquin G.J., Brandolin G. (2003). Structure of mitochondrial ADP/ATP carrier in complex with carboxyatractyloside. Nature.

[9] Ruprecht J.J., Hellawell A.M., Harding M., Crichton P.G., McCoy A.J., Kunji E.R. (2014). Structures of yeast mitochondrial ADP/ATP carriers support a domain-based alternating-access transport mechanism. Proc. Natl. Acad. Sci. USA.

[10] Palmieri F., Pierri C.L. (2010). Structure and function of mitochondrial carriers—Role of the transmembrane helix P and G residues in the gating and transport mechanism. FEBS Lett..

[11] Kunji E.R., Robinson A.J. (2010). Coupling of proton and substrate translocation in the transport cycle of mitochondrial carriers. Curr. Opin. Struct. Biol..

[12] Wang Y., Tajkhorshid E. (2008). Electrostatic funneling of substrate in mitochondrial inner membrane carriers. Proc. Natl. Acad. Sci. USA.

[13] Johnston J.M., Khalid S., Sansom M.S. (2008). Conformational dynamics of the mitochondrial ADP/ATP carrier: A simulation study. Mol. Membr. Biol..

[14] Dehez F., Pebay-Peyroula E., Chipot C. (2008). Binding of ADP in the mitochondrial ADP/ATP carrier is driven by an electrostatic funnel. J. Am. Chem. Soc..

[15] Ruprecht J.J., King M.S., Zogg T., Aleksandrova A.A., Pardon E., Crichton P.G., Steyaert J., Kunji E.R.S. (2019). The Molecular Mechanism of Transport by the Mitochondrial ADP/ATP Carrier. Cell.

[16] Mao X., Yao S., Yi Q., Xu Z.M., Cang X. (2021). Function-related asymmetry of the specific cardiolipin binding sites on the mitochondrial ADP/ATP carrier. Biochim. Biophys. Acta Biomembr..

[17] Yao S., Yi Q., Ma B., Mao X., Chen Y., Guan M.-X., Cang X. (2021). Structural Basis of Substrate Recognition by the Mitochondrial ADP/ATP Transporter. BioRxiv.

[18] Montalvo-Acosta J.J., Kunji E.R.S., Ruprecht J.J., Dehez F., Chipot C. (2021). Structure, substrate binding, and symmetry of the mitochondrial ADP/ATP carrier in its matrix-open state. Biophys. J..

[19] Falconi M., Chillemi G., Di Marino D., D’Annessa I., Morozzo della Rocca B., Palmieri L., Desideri A. (2006). Structural dynamics of the mitochondrial ADP/ATP carrier revealed by molecular dynamics simulation studies. Proteins.

[20] Ardalan A., Sowlati-Hashjin S., Oduwoye H., Uwumarenogie S.O., Karttunen M., Smith M.D., Jelokhani-Niaraki M. (2021). Biphasic Proton Transport Mechanism for Uncoupling Proteins. J. Phys. Chem..

[21] Nelson D.R., Lawson J.E., Klingenberg M., Douglas M.G. (1993). Site-directed mutagenesis of the yeast mitochondrial ADP/ATP translocator. Six arginines and one lysine are essential. J. Mol. Biol..

[22] Muller V., Basset G., Nelson D.R., Klingenberg M. (1996). Probing the role of positive residues in the ADP/ATP carrier from yeast. The effect of six arginine mutations of oxidative phosphorylation and AAC expression. Biochemistry.

[23] Thompson K., Majd H., Dallabona C., Reinson K., King M.S., Alston C.L., He L., Lodi T., Jones S.A., Fattal-Valevski A. (2016). Recurrent De Novo Dominant Mutations in SLC25A4 Cause Severe Early-Onset Mitochondrial Disease and Loss of Mitochondrial DNA Copy Number. Am. J. Hum. Genet..

[24] Mielke C., Lefort N., McLean C.G., Cordova J.M., Langlais P.R., Bordner A.J., Te J.A., Ozkan S.B., Willis W.T., Mandarino L.J. (2014). Adenine Nucleotide Translocase Is Acetylated in Vivo in Human Muscle: Modeling Predicts a Decreased ADP Affinity and Altered Control of Oxidative Phosphorylation. Biochemistry.

[25] Finlayson J., Barakati N., Langlais P.R., Funk J., Zapata Bustos R., Coletta D.K., Luo M., Willis W.T., Mandarino L.J. (2021). Site-specific acetylation of adenine nucleotide translocase 1 at lysine 23 in human muscle. Anal. Biochem..

[26] Bertholet A.M., Chouchani E.T., Kazak L., Angelin A., Fedorenko A., Long J.Z., Vidoni S., Garrity R., Cho J., Terada N. (2019). H(+) transport is an integral function of the mitochondrial ADP/ATP carrier. Nature.

[27] Delano W. (2002). Pymol Molecular Graphics System: An open-source molecular graphics tool. CCP4 Newsl. Protein Crystallogr..

[28] Hess B., Kutzner C., van der Spoel D., Lindahl E. (2008). GROMACS 4: Algorithms for Highly Efficient, Load-Balanced, and Scalable Molecular Simulation. J. Chem. Theory Comput..

[29] Huang J., MacKerell A.D. (2013). CHARMM36 all-atom additive protein force field: Validation based on comparison to NMR data. J. Comput. Chem..

[30] MacKerell A., Feig M., Rd B.C. (2004). Extending the treatment of backbone energetics in protein force fields: Limitations of gas-phase quantum mechanics in reproducing protein conformational distributions in molecular dynamics simulations. J. Comput. Chem..

[31] Klauda J.B., Venable R.M., Freites J.A., O’Connor J.W., Tobias D.J., Mondragon-Ramirez C., Vorobyov I., MacKerell A.D., Pastor R.W. (2010). Update of the CHARMM all-atom additive force field for lipids: Validation on six lipid types. J. Phys. Chem..

[32] Bussi G., Donadio D., Parrinello M. (2007). Canonical sampling through velocity rescaling. J. Chem. Phys..

[33] Berendsen H.J.C., Postma J.P.M., Vangunsteren W.F., Dinola A., Haak J.R. (1984). Molecular-Dynamics with Coupling To an External Bath. J. Chem. Phys..

[34] Miyamoto S., Kollman P.A. (1992). Settle—An Analytical Version of the Shake and Rattle Algorithm for Rigid Water Models. J. Comput. Chem..

[35] Hess B., Bekker H., Berendsen H.J.C., Fraaije J.G.E.M. (1997). LINCS: A linear constraint solver for molecular simulations. J. Comput. Chem..

[36] Essmann U., Perera L., Berkowitz M.L., Darden T., Lee H., Pedersen L.G. (1995). A smooth particle mesh Ewald method. J. Chem. Phys..

[37] Humphrey W.F., Dalke A., Schulten K. (1996). VMD: Visual molecular dynamics. J. Mol. Graph..

