# Supplementary material for: Investigating the Broad Matrix-Gate Network in the Mitochondrial ADP/ATP Carrier through Molecular Dynamics Simulations
Source: Molecules. 2022 Feb 5;27(3):1071. doi: 10.3390/molecules27031071 (PMC8839422; doi:10.3390/molecules27031071)
Supplement: Supplementary file 1 [file molecules-27-01071-s001.zip › molecules-1576510-supplementary.pdf]

Supplementary Materials for

Investigating the broad matrix-gate network in the mitochondrial ADP/ATP carrier through molecular dynamics simulations

Shihao Yao[1,2], Boyuan Ma[1,2], Qiuzi Yi[1,2], Min-Xin Guan[1,2,*], Xiaohui Cang[1,2,*]

*Corresponding authors: xhcang@zju.edu.cn (X.C.) or to: gminxin88@zju.edu.cn (M.-X. G.)

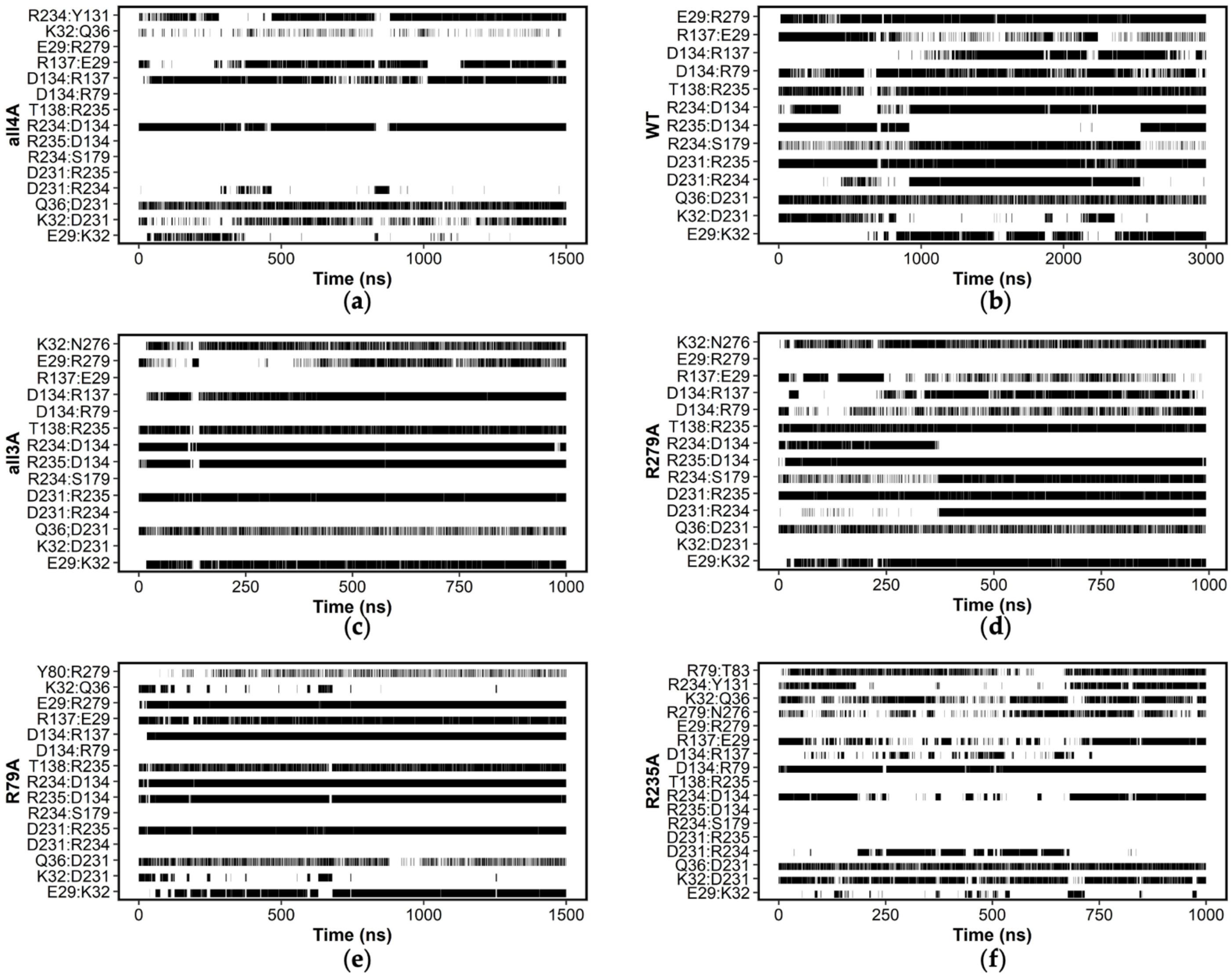

Supplementary Figure S1. The time evolution of the electrostatic interactions within the broad m-gate network in MD simulations on wild-type **(a)** and mutant **(b-f)** AAC.

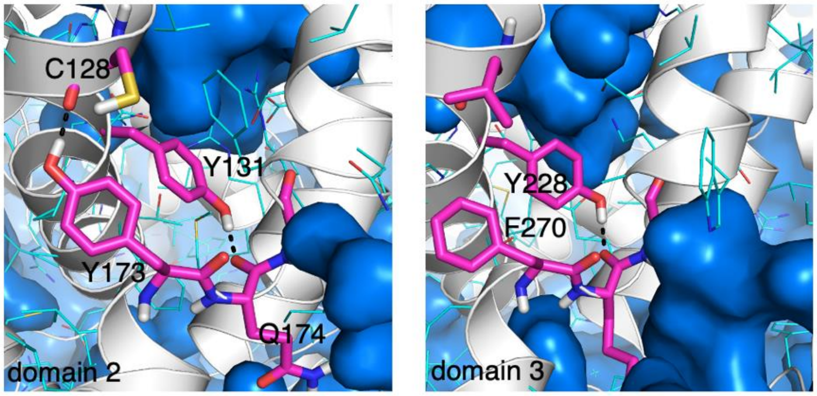

(a) (b)

Supplementary Figure S2. The solvent within the pocket and bulk solvent at the matrix side is more separated due to presence of a tyrosine before the kink proline in domain 2 (**a**) and domain 3 (**b**).

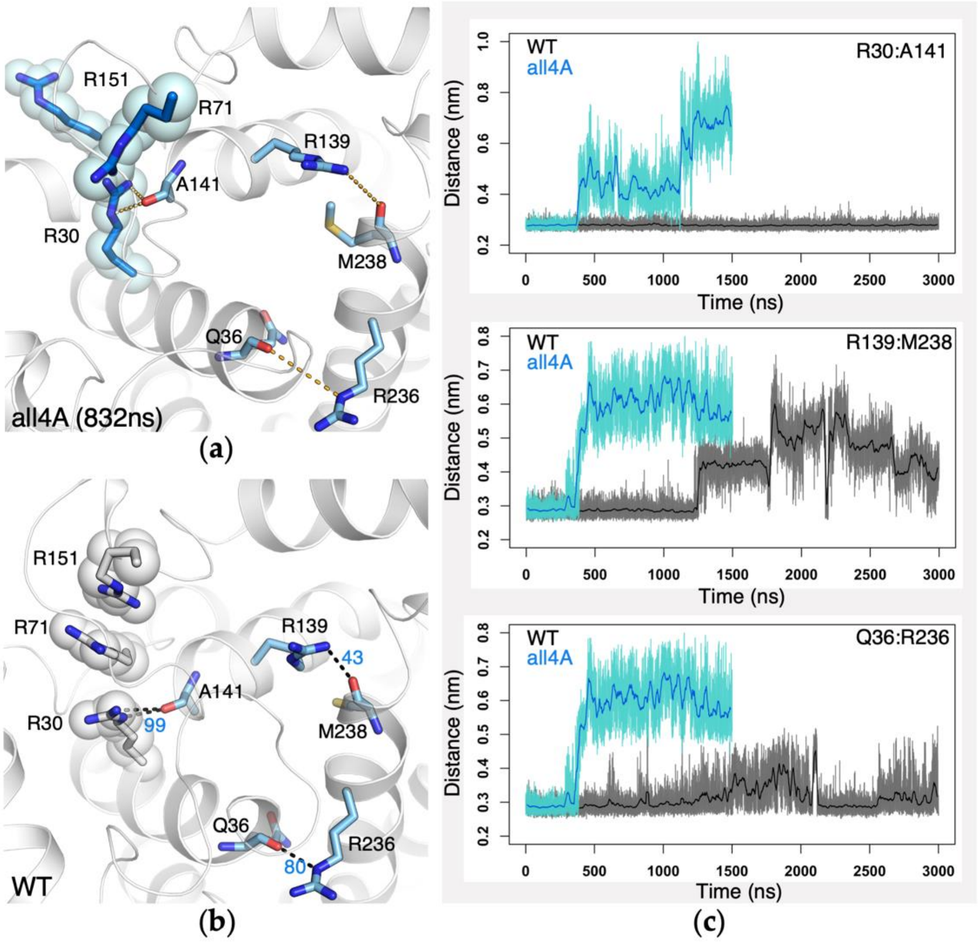

Supplementary Figure S3. The R30:R71:R151 stacking structure and time evolution of electrostatic interactions between the capping arginines and N-termini of odd-numbered helices. (**a**) A snapshot of *all4A-AAC* in which the the R30:R71:R151 stacking structure became dissociated and the cyclic electrostatic network between capping arginines (R30, R139 and R236) and N-termini of odd-numbered helices is disrupted. Yellow dash lines are added manually to indicate the H-bonds that do not appear in the shown snapshot. (**b**) A snapshot of *wild-type* AAC in which the the R30:R71:R151 stacking structure and the cyclic electrostatic network between capping arginines and N-termini of odd-numbered helices are maintained. The cyclic electrostatic interactions are shown in black dash lines, with occupancies shown in blue numbers. The occupancies were calculated over the 3-μs trajectory. (**c**) Time evolutions of the distances between the three capping arginines and the corresponding N-termini of odd-numbered helices.
